# The Interplay between NF-kappaB and E2F1 Coordinately Regulates Inflammation and Metabolism in Human Cardiac Cells

**DOI:** 10.1371/journal.pone.0019724

**Published:** 2011-05-23

**Authors:** Xavier Palomer, David Álvarez-Guardia, Mercy M. Davidson, Tung O. Chan, Arthur M. Feldman, Manuel Vázquez-Carrera

**Affiliations:** 1 Department of Pharmacology and Therapeutic Chemistry, IBUB (Institut de Biomedicina de la Universitat de Barcelona) and CIBERDEM, Faculty of Pharmacy, University of Barcelona, Barcelona, Spain; 2 Department of Radiation Oncology, Columbia University, New York, New York, United States of America; 3 Department of Medicine, Center for Translational Medicine, Thomas Jefferson University, Philadelphia, Pennsylvania, United States of America; University of Colorado Denver, United States of America

## Abstract

Pyruvate dehydrogenase kinase 4 (PDK4) inhibition by nuclear factor-κB (NF-κB) is related to a shift towards increased glycolysis during cardiac pathological processes such as cardiac hypertrophy and heart failure. The transcription factors estrogen-related receptor-α (ERRα) and peroxisome proliferator-activated receptor (PPAR) regulate *PDK4* expression through the potent transcriptional coactivator PPARγ coactivator-1α (PGC-1α). NF-κB activation in AC16 cardiac cells inhibit ERRα and PPARβ/δ transcriptional activity, resulting in reduced *PGC-1α* and *PDK4* expression, and an enhanced glucose oxidation rate. However, addition of the NF-κB inhibitor parthenolide to these cells prevents the downregulation of *PDK4* expression but not ERRα and PPARβ/δ DNA binding activity, thus suggesting that additional transcription factors are regulating *PDK4*. Interestingly, a recent study has demonstrated that the transcription factor E2F1, which is crucial for cell cycle control, may regulate *PDK4* expression. Given that NF-κB may antagonize the transcriptional activity of E2F1 in cardiac myocytes, we sought to study whether inflammatory processes driven by NF-κB can downregulate *PDK4* expression in human cardiac AC16 cells through E2F1 inhibition. Protein coimmunoprecipitation indicated that *PDK4* downregulation entailed enhanced physical interaction between the p65 subunit of NF-κB and E2F1. Chromatin immunoprecipitation analyses demonstrated that p65 translocation into the nucleus prevented the recruitment of E2F1 to the *PDK4* promoter and its subsequent E2F1-dependent gene transcription. Interestingly, the NF-κB inhibitor parthenolide prevented the inhibition of E2F1, while E2F1 overexpression reduced interleukin expression in stimulated cardiac cells. Based on these findings, we propose that NF-κB acts as a molecular switch that regulates E2F1-dependent *PDK4* gene transcription.

## Introduction

Enlargement of the mammalian heart occurs principally by cell hypertrophy, since post-natal cardiac myocytes lack the ability to undergo cell division. This adaptive physiological growth allows the heart to maintain sufficient cardiac output. Myocardial injury owing to myocardial infarction or chronic hypertension leads to pathological hypertrophic growth that may result in heart failure. Tumor necrosis factor (TNF)-α is a pro-inflammatory cytokine secreted by the myocardium that has been related to cardiac hypertrophy and chronic heart failure [1], [2]. Inflammatory cytokines are under the transcriptional control of the ubiquitous inducible factor named nuclear factor-κB (NF-κB), which has been linked to various cardiovascular diseases, such as cardiac hypertrophy and heart failure [3].

The development of pathological cardiac hypertrophy occurs together with re-activation of the cell cycle machinery of myocytes [4]. The transcription factor E2F1 is one of the key proteins in the regulation of the G1/S phase transition, hence it acts as a critical regulator of cell survival and proliferation [4]. However, E2F1 activity has antagonistic functions, since it may induce cell proliferation or apoptosis [5]. To form functional transcription complexes on DNA, E2F1 requires heterodimerization with the partner differentiation regulated transcription factor (DRTF) polypeptide DP-1. The transcriptional activity of E2F1 is sterically inhibited by the retinoblastoma protein (pRB) family of pocket proteins [6]. When pRB proteins are phosphorylated, the E2F-DP complex is released, hence E2F-mediated gene transcription commences.

The heart can adapt to various pathophysiological conditions by adjusting its relative metabolism of carbohydrates and fatty acids. Consequently, loss of this metabolic flexibility is associated with cardiovascular disease. Metabolic changes in cardiac substrate utilization entail the dysregulation of genes involved in the transport and catabolism of fatty acids and glucose. The transcription factors estrogen-related receptor-α (ERRα) and peroxisome proliferator-activated receptor (PPAR) regulate the pyruvate dehydrogenase kinase 4 (PDK4) expression, a key enzyme in glucose homeostasis modulation [7], [8]. In particular, they regulate *PDK4* expression through the potent transcriptional coactivator PPARγ coactivator-1α (PGC-1α) [9], [10]. A previous study performed in our laboratory revealed that NF-κB activation in cardiac cells inhibited ERRα and PPARβ/δ DNA binding activity, which resulted in reduced *PGC-1α and PDK4* expression, and an enhanced glucose oxidation rate [11]. However, addition of the NF-κB inhibitor parthenolide prevented the downregulation of *PGC-1α and PDK4* expression but not ERRα and PPARβ/δ DNA binding activity [11]. Besides, addition of this NF-κB inhibitor in the absence of TNF-α to human cardiac AC16 cells [11] or neonatal rat cardiomyocytes [12] induces *PDK4* expression to levels that far exceed those observed at the basal state. Importantly, this induction is not correlated with an upregulation in *PGC-1α* expression or ERRα-PPARβ/δ transcriptional activity. These findings reinforce the notion that additional PGC-1α-independent transcription factors regulate *PDK4* to keep cardiac cell metabolism within a balanced physiological margin in these cells. Interestingly, recent studies report that E2F1 may regulate other genes besides those involved in cell-cycle regulation [13], [14]. For instance, loss of E2F1 in vivo blunts PDK4 expression, while exogenous E2F1 overexpression up-regulates PDK4 levels in mouse myoblasts and IMR90 fibroblasts [14]. Such effects are driven by the binding of E2F1 to the E2F binding sites located within the promoter of the gene that encodes for *PDK4*. On the other hand, other studies demonstrate that NF-κB may antagonize the transcriptional activity of E2F1 in cardiac myocytes [15] and human fibroblasts [16]. Therefore, since NF-κB may antagonize the transcriptional activity of E2F1, and E2F1 is able to regulate PDK4, the present study aimed to elucidate whether E2F1 was involved in the downregulation of PDK4 expression induced by NF-κB activation in cardiac myocytes.

## Results

### Inhibition of PDK4 expression by TNF-α coincides with dysregulation of E2F1 activity in AC16 cells

**Figure 1:**
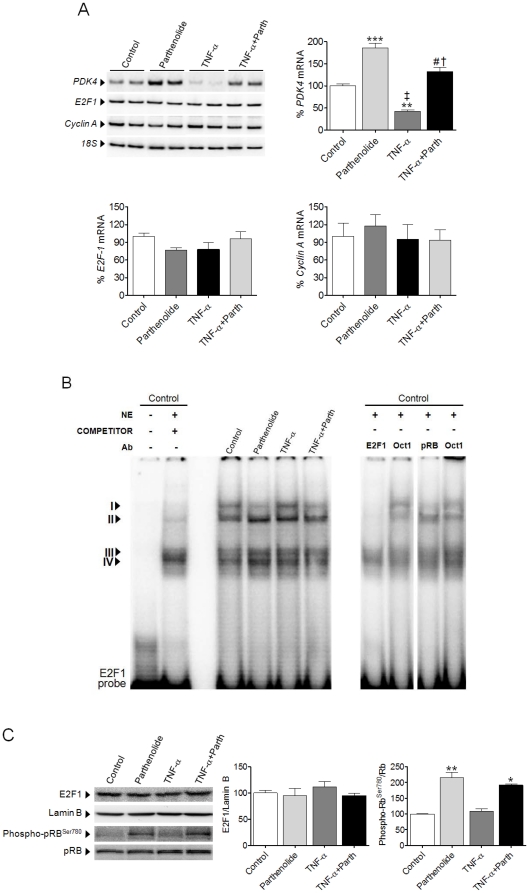
NF-κB modulation influences E2F1 activity in human cardiac cells. (**A**) Relative quantification of *PDK4*, *E2F1* and *Cyclin A* mRNA levels assessed by RT-PCR in human cardiac AC16 cells incubated with TNF-α (100 ng/mL) for 24 h in the presence or absence of parthenolide (Parth, 10 µmol/L). The graphics represent the quantification of the *18S*-normalized mRNA levels, expressed as a percentage of control samples ±STD. (**B**) EMSA assay showing E2F1 DNA-binding activity after treatment of AC16 cells with TNF-α in the presence or absence of parthenolide (NE, nuclear extracts; Ab, antibody). (**C**) E2F1, phospho-pRB^Ser780^ and total pRB protein levels in nuclear protein extracts isolated from samples as described in panel A. To show equal loading of protein, the Lamin B signal is also included. The graphics represent the quantification of the normalized protein levels, expressed as a percentage of control samples ±STD. All autoradiograph data are representative of three separate experiments. *P<0.05, **P<0.01, ***P<0.001 vs. Control; †P<0.01, ‡ P<0.001 vs. Parth; # P<0.001 vs. TNFα.

Addition of TNF-α (100 ng/mL for 24 h) to AC16 cells inhibited *PDK4* expression (∼60% reduction, P<0.01, **Figure 1A**). Parthenolide not only prevented this, but was also capable of inducing *PDK4* mRNA to levels beyond those observed in non-stimulated cells. As a first approach, we investigated whether these changes correlated with dysregulation of the E2F1 signaling pathway. No changes in *E2F1* expression were observed after treatment with TNF-α for 6 h (see Supplementary **Figure S1A**) or 24 h (**Figure 1A**). *Cyclin A*, whose expression is induced by E2F1 [17], was not modified in these conditions. Nevertheless, E2F1 DNA-binding activity displayed some changes when examined by means of an EMSA (**Figure 1B**). E2F1 formed four DNA-binding complexes, namely I to IV, with nuclear proteins. However, the competitor lane demonstrated that only complexes I to III were specific for the E2F1 probe, while complex IV corresponded to a non-specific band. Supershift analyses demonstrated that complexes I and II contained the E2F1 transcription factor, while pRB was exclusively present in complex I. Thus, complex I might correspond to a complex containing the E2F1-DP heterodimer along with pocket proteins such as pRB, while complexes II and III might represent the free E2F1-DP. Independently of the presence of TNF-α, parthenolide downregulated complex I. This NF-κB inhibitor also increased complex II, particularly in the absence of TNF-α. This suggests that the levels of pRB bound to E2F1 were downregulated by parthenolide. In contrast, treatment with TNF-α enhanced the DNA-binding of complexes I and II (**Figure 1B**). We next evaluated the protein levels of E2F1 and pRB. E2F1 remained unaltered in nuclear protein extracts from AC16 cells treated with TNF-α for 6 h (see Supplementary **Figure S1B**) or 24 h (**Figure 1C**). The addition of parthenolide did not alter E2F1 protein levels either, although it significantly enhanced the phosphorylation of pRB at serine 780.

### PDK4 expression is regulated by E2F1 in AC16 cells

**Figure 2:**
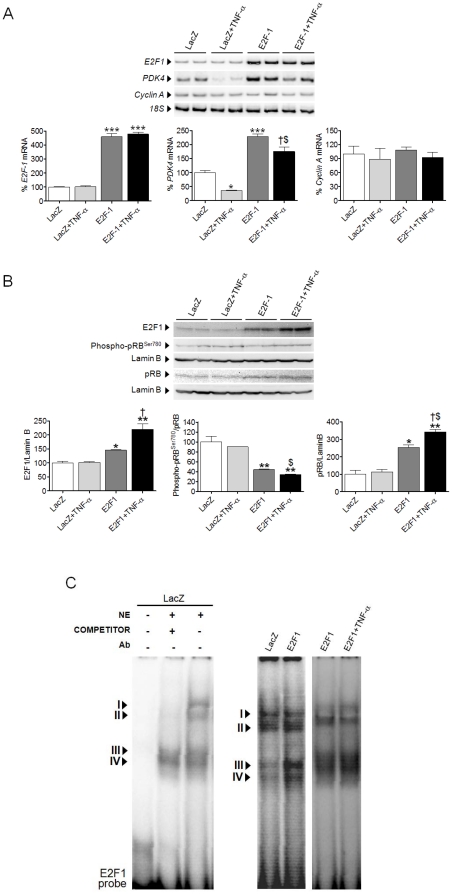
E2F1 overexpression induces *PDK4* transcription in human cardiac AC16 cells. (**A**) Relative quantification of *E2F1*, *PDK4* and *cyclin A* mRNA levels assessed by RT-PCR in human cardiac AC16 cells treated with or without TNF-α (100 ng/ml, 24 h) and transfected with LacZ- or E2F1-carrying plasmids, as described in Figure 1A. (**B**) E2F1, phospho-pRB^Ser780^ and total pRB protein levels in nuclear protein extracts. To show equal loading of protein, the Lamin B signal from the same blot is included. The graphics represent the quantification of the normalized protein levels, expressed as a percentage of control samples ±STD. (**C**) EMSA assay showing E2F1 DNA-binding activity after treatment of AC16 cells as described in panel A (NE, nuclear extracts; Ab, antibody). All autoradiograph data are representative of three separate experiments. *P<0.05, **P<0.01, ***P<0.001 vs. LacZ; †P<0.05 vs. E2F1; $P<0.01 vs. LacZ+TNF-α.

**Figure 3:**
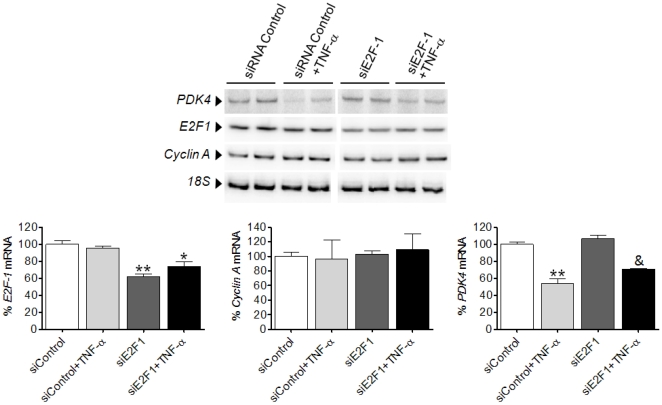
*E2F1* gene silencing does not enhance the TNF-α-mediated PDK4 inhibition. Relative quantification of *E2F1*, *PDK4* and *cyclin A* mRNA levels assessed by RT-PCR in human cardiac AC16 cells treated with or without TNF-α and transfected with scrambled siRNA (siControl) or siE2F1. The graphics represent the quantification of the *18S*-normalized mRNA levels, expressed as a percentage of control samples ±STD. All autoradiograph data are representative of three separate experiments. *P<0.05, **P<0.01 vs. siRNA Control; &P<0.01 vs. siE2F1.

E2F1 overexpression significantly enhanced *E2F1* mRNA levels (**Figure 2A**) in human AC16 cells, even in the presence of TNF-α, compared to control cells transfected with a lacZ-containing plasmid. Despite the huge increment in *E2F1* expression, *cyclin A* was not modified. In contrast, *PDK4* expression correlated with E2F1 mRNA levels, which demonstrates that this kinase is transcriptionally induced by E2F1 in cardiac AC16 cells (**Figure 2A**). Furthermore, overexpression of E2F1 partially counteracted TNF-α-induced *PDK4* downregulation in these cells. Forced *E2F1* expression in AC16 cells induced a significant increase in E2F1 protein accumulation (**Figure 2B**). Strikingly, this rise was further enhanced in nuclear protein extracts when TNF-α was added to the medium. E2F1 levels correlated with those of total pRB protein, while the ratio phospho-pRB^Ser780^/pRB was reduced in cells overexpressing E2F1 (**Figure 2B**). Overexpression of *E2F1* enhanced the DNA-binding activity of complexes II and III of E2F1 in comparison to the control cells (**Figure 2C**), although TNF-α did not alter this activity. The overall results might explain why *PDK4* expression was not enhanced by TNF-α in cells overexpressing E2F1 when compared to cells lacking the stimulus, in spite of the increase of E2F1 protein. Subsequently, small interfering RNA (siRNA)-mediated *E2F1* gene silencing was carried out by transfecting AC16 cells with human *E2F1* siRNA. A reduction of up to 40% in *E2F1* expression by means of siE2F1 did not reduce *PDK4* or *cyclin A* expression as compared to control cells transfected with scrambled siRNA (**Figure 3**).

### TNF-α enhances the physical interaction between the p65 subunit of NF-κB and E2F1 in human and mouse cardiac cells

**Figure 4:**
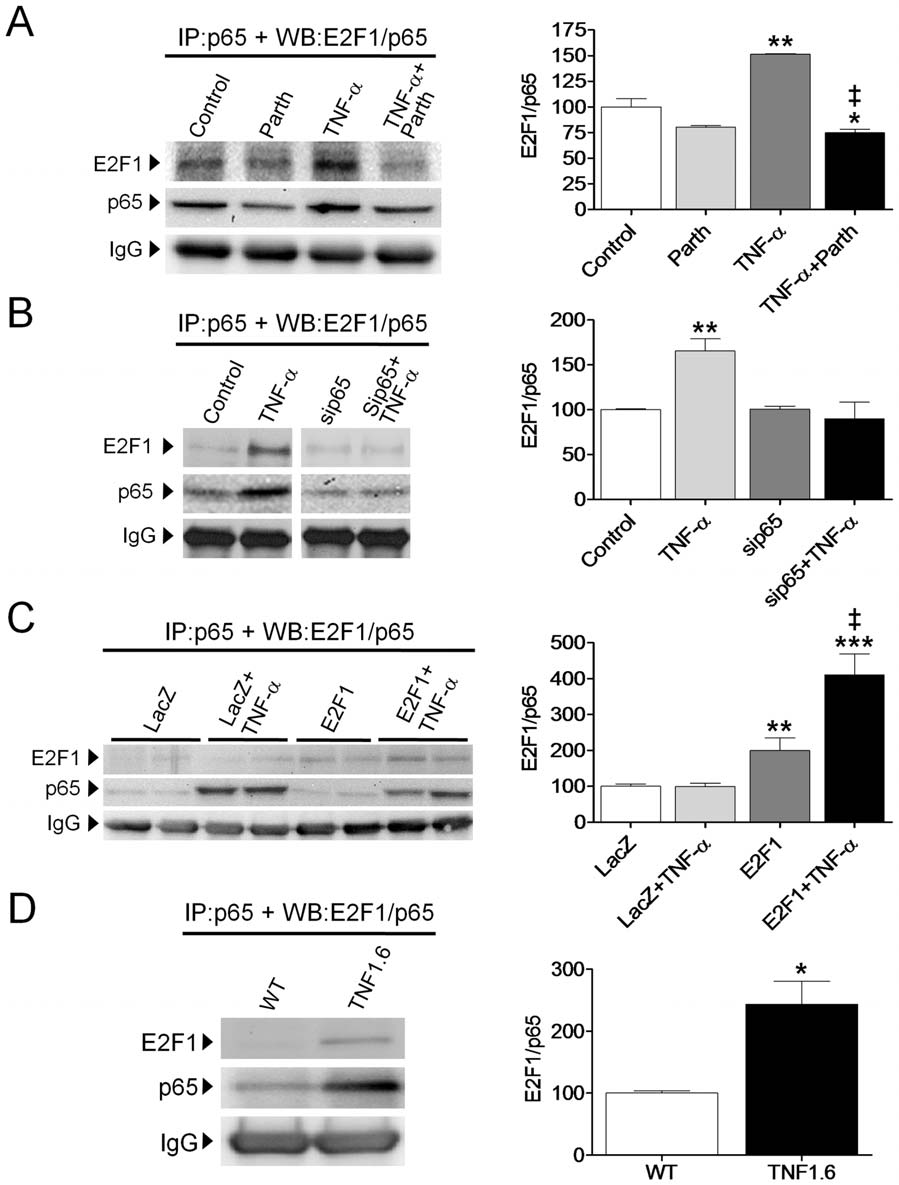
p65 subunit of NF-κB and E2F1 are physically associated in cardiac cells. Protein extracts were immunoprecipitated using an anti-p65 antibody and then subjected to SDS-PAGE and immunoblotted with an anti-E2F1 antibody. The graphics represent the quantification of the immunoprecipitated protein normalized to its corresponding control samples, expressed as a percentage ±STD. The blot data are representative of three separate experiments. (**A**) AC16 cells treated with TNF-α (100 ng/ml, 24 h) in the presence or absence of parthenolide (Parth, 10 µmol/L). (**B**) AC16 cells treated with or without TNF-α and transfected with specific siRNA to *p65*. (**C**) AC16 cells treated with or without TNF-α and transfected with LacZ- or E2F1-carrying plasmids. (**D**) TNF1.6 or control wild type (WT) mice. (**A**) and (**B**) *P<0.05, **P<0.01 vs. Control; ‡P<0.01 vs. TNFα. (**C**) *P<0.05, **P<0.01, ***P<0.001 vs. LacZ; ‡P<0.001 vs. E2F1. (**D**) *P<0.05 vs. WT.

In non-stimulated mammalian cells, NF-κB mostly consists of an inactive heterodimeric complex comprised of p50 and p65 subunits sequestered in the cytoplasm by association with the inhibitory IκB protein. In response to proinflammatory cytokines, IκB is phosphorylated by the IκB kinase (IKK) complex, which leads to its degradation by the proteasome. Once IκB is degraded, NF-κB translocates into the nucleus where it binds to specific promoter sites on its target genes. However, NF-κB may also localize to the nucleus in non-stimulated cells, where it would act as a transcriptional repressor [15]. Since NF-κB may associate with E2F, we next investigated whether this mechanism might be involved in the regulation of *PDK4* expression in cardiac cells. Coimmunoprecipitation studies revealed that p65 was constitutively bound to E2F1 in resting cells, and this binding was increased upon NF-κB activation with TNF-α (**Figure 4A**). The opposite behavior was observed after parthenolide addition. Next, we investigated the effects of knocking down *p65* with a specific siRNA (sip65). Transfection with sip65 downregulated the levels of this protein by up to 30% when compared to control siRNA [18], and this reduction was sufficient to prevent the enhanced interaction of p65 with E2F1 induced by TNF-α (**Figure 4B**). Then we examined the effect of E2F1 protein overexpression on its association with the p65 subunit. As shown in the graph in **Figure 4C**, overexpression of E2F1 increased the p65-E2F1 association (2-fold, P<0.01 vs. LacZ), which was further enhanced when TNF-α was added to E2F1-transfected cells (4-fold, P<0.001 vs. LacZ). In contrast, E2F1 gene silencing by means of siRNA did not influence the binding between E2F1 and p65 proteins (data not shown).

To further confirm the results obtained in vitro, we performed studies with mice. Transgenic TNF1.6 mice, which present reduced *PDK4* expression in the heart compared with wild-type mice (see Supplementary **Figure S2A**), also displayed enhanced binding of p65 to E2F1 in heart (**Figure 4D**). Analyses of *E2F1* and *cyclin A* mRNA, as well as E2F1 protein levels, showed no differences between wild-type and transgenic TNF1.6 mice (Supplementary **Figure S2A and B**).

### The physical interaction between p65 and E2F1 results in a diminution of the binding of E2F1 to the PDK4 promoter

**Figure 5:**
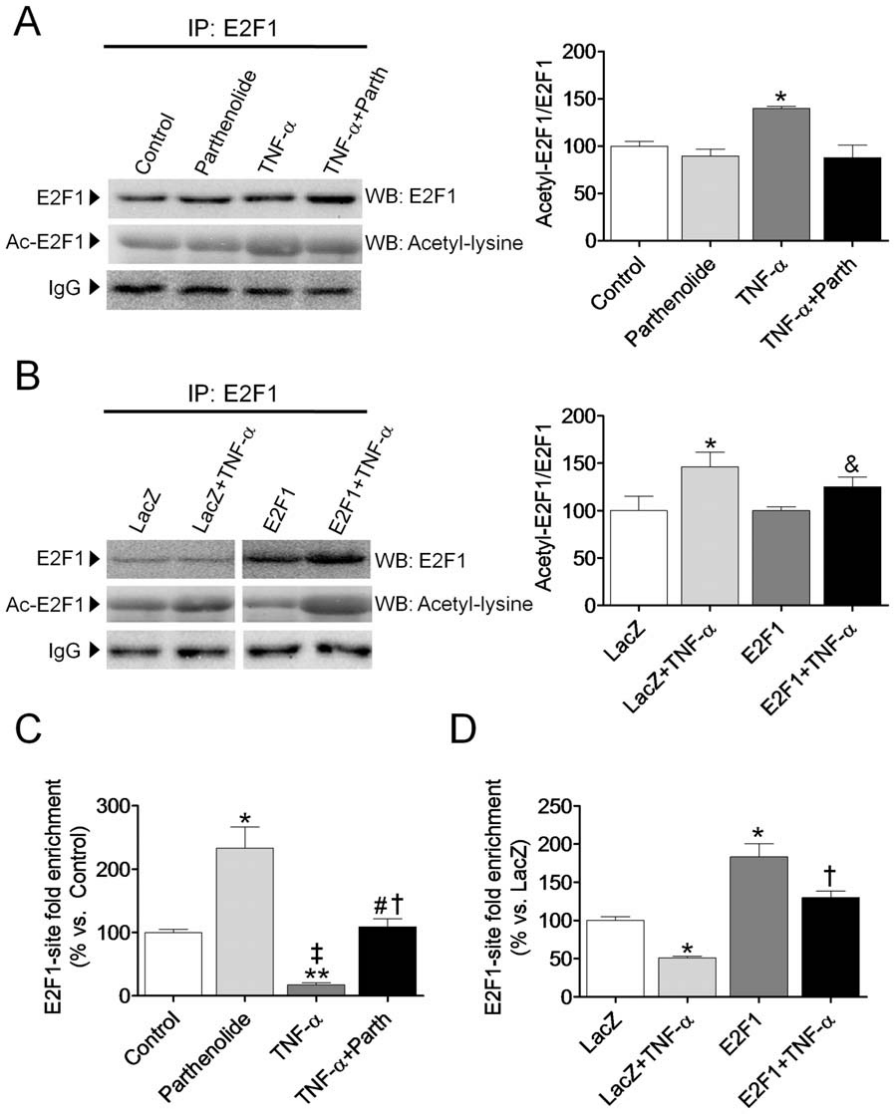
TNF-α inhibits *PDK4* promoter occupancy by E2F1. (**A**) and (**B**), protein extracts of AC16 cells treated with TNF-α (100 ng/mL, 24 h) in the presence or absence of 10 µmol/L parthenolide (**A**) or transfected with LacZ- or E2F1-carrying plasmids (**B**) were subjected to immunoprecipitation using an anti-E2F1 antibody. They were then subjected to SDS-PAGE and immunoblotted with an anti-acetyl-lysine antibody. The graphics represent the quantification of the immunoprecipitated protein normalized to its corresponding control samples, expressed as a percentage ±STD. The blot data are representative of three separate experiments. (**C**) and (**D**), chromatin immunoprecipitation from AC16 cells incubated with TNF-α in the presence or absence of parthenolide (**C**); or AC16 cells treated with TNF-α and transfected with LacZ- or E2F1-carrying plasmids (**D**). The graphics represent the E2F1-site fold enrichment at the PDK4 promoter, expressed as a percentage of control (**C**) or LacZ (**D**) samples ±STD. (**A**) and (**C**) *P<0.05, **P<0.01 vs. Control; †P<0.05, ‡P<0.01 vs. Parth; # P<0.05 vs. TNFα. (**B**) and (**D**) *P<0.05 vs. LacZ; †P<0.05 vs. LacZ+TNF-α; &P<0.05 vs. E2F1.

Retinoblastoma family proteins (pRB, p107 and p130) inhibit E2F transcriptional activity by disrupting histone acetyltransferase binding and the recruitment of histone deacetylases. Since we observed that parthenolide induced the phosphorylation of pRB at Ser780, the release of E2F1 and its subsequent acetylation might account for the enhanced *PDK4* expression in AC16 cells. Therefore, acetylation of E2F1 was examined by means of coimmunoprecipitation analysis. E2F1 acetylation was not modified after NF-κB inhibition with parthenolide. In fact, it appeared to be enhanced by TNF-α (**Figure 5A**). These results were further confirmed when E2F1 acetylation was examined in AC16 cells overexpressing E2F1 (**Figure 5B**). Thus, we hypothesize that enhanced acetylation was not involved in *PDK4* upregulation, but it might be a response of the cell to counteract the TNF-α-induced inhibition of E2F1.

Subsequently, a ChIP assay was employed to determine whether E2F1 is recruited to the promoter of the *PDK4* gene in cardiac AC16 cells. This analysis showed that the specific anti-E2F1 antibody, but not the non-immune IgG control, successfully co-immunoprecipitated E2F1 and significant quantities of *PDK4* promoter under basal conditions (**Figure 5C**). **Figure 5C** also reveals that the E2F1 antibody pulled down significantly more of the *PDK4* promoter when the NF-κB inhibitor parthenolide was added to the medium. In contrast, E2F1-binding to the *PDK4* promoter region was largely reduced in the presence of TNF-α. In support of a role for E2F1 in the transcriptional control of *PDK4*, AC16 cells transfected with the pSG5L/E2F1 plasmid displayed enhanced PDK4 promoter occupancy by E2F1 (**Figure 5D**). The negative effect of NF-κB activation by TNF-α on the PDK4 promoter occupancy by E2F1 was further corroborated in cells overexpressing E2F1 (**Figure 5D**).

### The crosstalk between the p65 subunit of NF-κB and E2F1 influences TNF-α-induced inflammation and glucose oxidation in human cardiac cells

**Figure 6:**
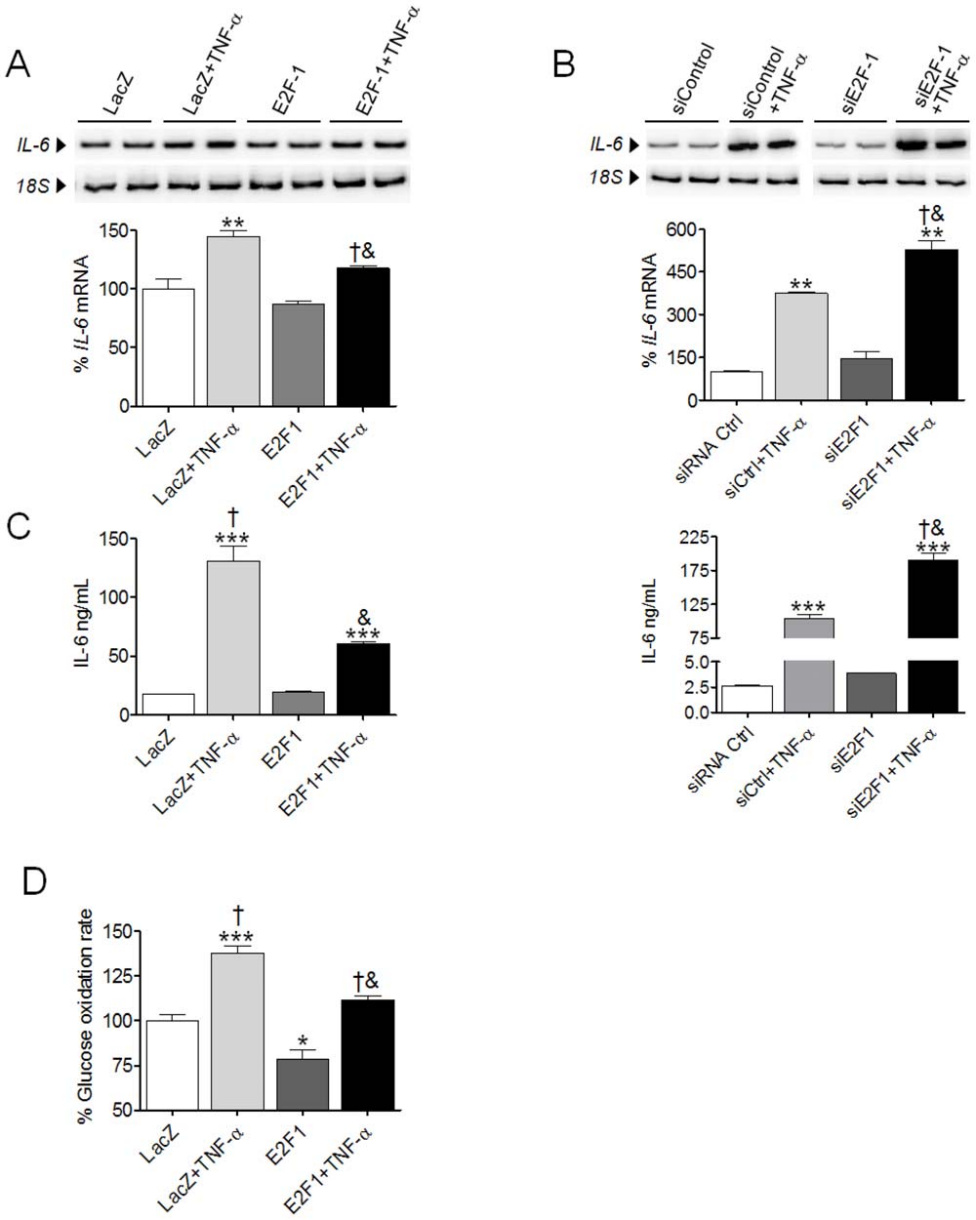
The crosstalk between the p65 subunit of NF-κB and E2F1 influences inflammation and glucose oxidation. (**A**) and (**B**), Relative quantification of *IL-6* mRNA levels assessed by RT-PCR in human cardiac AC16 cells transfected with LacZ- or E2F1-carrying plasmids (**A**) or with control siRNA (siCtrl) and siE2F1 (**B**) and treated with or without TNF-α (100 ng/ml) for 8 h (**A**) or 24 h (**B**). Graphics represent the quantification of the *18S*-normalized mRNA levels, expressed as a percentage of control samples ±STD. The blot data are representative of three separate experiments. (**C**) Levels of IL-6 (ng/mL) in the same samples as described in Panels A and B. (**D**) [U-^14^C]-glucose oxidation rates in AC16 cells overexpressing the human *LacZ*-control or the *E2F1* genes incubated with TNF-α. (**A**), (**C**), and (**D**) *P<0.05, **P<0.01, ***P<0.001 vs. LacZ; †P<0.01 vs. E2F1; &P<0.01 vs. LacZ+TNF-α. (**B**) *P<0.05, **P<0.01, ***P<0.001 vs. siCtrl; †P<0.001 vs. siCtrl+TNF-α; & P<0.001 vs. siE2F1.

Gene expression analyses of *IL-6* in cardiac AC16 cells carrying pSG5L/E2F1 revealed that this transcription factor might inhibit NF-κB activity in the presence of a proinflammatory stimulus. Thus, the mRNA for *IL-6* was partially inhibited by E2F1 overexpression in the presence of TNF-α when compared to control LacZ samples (**Figure 6A**). In contrast, *IL-6* mRNA was upregulated when *E2F1* expression was downregulated by siRNA technology (**Figure 6B**). The levels of IL-6 secreted into the medium correlated with the expression of the gene in both cases (**Figure 6C**).

Finally, overexpression of the human E2F1 protein reduced the glucose oxidation rate (∼25% reduction, P<0.05, **Figure 6D**). Addition of TNF-α to these E2F1-transfected cells increased the catabolism of glucose (75% vs. 115%), although the increase observed in control LacZ-transfected cells treated with TNF-α was not reached (**Figure 6D**).

## Discussion

The progression of heart failure usually entails a local rise in pro-inflammatory cytokines, such as TNF-α, which mainly act in an autocrine fashion. In cardiac cells exposed to TNF-α, the inhibition of *PDK4* expression by NF-κB is related to the shift towards increased glycolysis that is observed during cardiac pathological processes induced by pro-inflammatory stimuli, such as cardiac hypertrophy and heart failure [11]. A previous study performed in our laboratory revealed that NF-κB activation in cardiac cells inhibited ERRα and PPARβ/δ DNA binding activity, which resulted in reduced *PDK4* expression and an enhanced glucose oxidation rate [11]. Addition of the NF-κB inhibitor parthenolide prevented the downregulation of *PDK4* expression but not ERRα and PPARβ/δ DNA binding activity, thus suggesting an additional mechanism by which *PDK4* is transcriptionally regulated. Besides ERRα and PPAR, a plethora of different transcription factors have been proposed to regulate *PDK4* expression, such as FOXO1 (Forkhead box protein O1), HNF4 (hepatic nuclear factor 4), LXR (liver×receptor) or RXR (retinoid×receptor) [7], [8]. For instance, glucocorticoids stimulate *PDK4* transcription in McA-RH7777 hepatoma cells through two glucocorticoid receptor binding sites located within the distal promoter region of the *PDK4* gene [19]. Interestingly, PGC-1α does not appear to be necessary for the acute regulation of *PDK4* by glucocorticoids [19]. Nevertheless, we did not found any change in FOXO1 activity in human cardiac AC16 cells treated with TNF-α (data not shown), thus ruling out this transcription factor as a regulator of *PDK4* expression in our conditions. For the first time, we propose a novel mechanism by which the inflammatory processes driven by NF-κB can downregulate PDK4 through inhibition of the E2F1 transcription factor in a PPAR- and ERRα-independent manner. The results demonstrate that inhibition of *PDK4* expression by TNF-α in cardiac AC16 cells coincides with dysregulation in E2F1 activity. In addition, we show that E2F1 can transcriptionally regulate the *PDK4* gene. E2F1 overexpression did not completely keep AC16 cells away from the downregulation in *PDK4* transcription observed after TNF-α addition, while silencing *E2F1* through siRNA technology had no effect on *PDK4* expression either. In consonance with this, enforced *PDK4* expression did not completely suppress glucose oxidation in human AC16 cells. These data indicate that additional regulatory mechanisms control *PDK4* transcription and glucose metabolism in cardiac cells, to prevent the deleterious effects of unrestrained E2F1 activities. Previous studies have already reported a link between E2F1 and glucose oxidation in muscle through the transcriptional regulation of *PDK4* expression [14]. This direct interaction between E2F1 and PDK4 might be aimed at sparing the glycolysis end-product pyruvate for the synthesis of the lipid and protein intermediates needed for cell doubling. E2F1 itself stimulates 6-phosphofructo-2-kinase-fructose-2,6-bisphosphatase, a potent stimulator of glycolysis [20].

The activity and specificity of E2F1 is tightly regulated through its gene expression and subcellular localization, as well as its interaction with pRB, phosphorylation or acetylation. These mechanisms of control do not act in isolation in time and space. We found that NF-κB activity modulation did not change E2F1 mRNA and protein levels in non-transfected AC16 cells. However, TNF-α significantly raised the E2F1 protein levels in cells carrying the pSG5L/E2F1 construct compared to non-stimulated cells. There is no simple explanation for this anomalous E2F1 accumulation, since it was not observed in control LacZ-transfected cells. However, increased protein stability owing to its interaction with p65 cannot be excluded. Regardless of the reason for the accumulation, the occurrence of higher levels of pRB concomitantly with a reduction in phospho-pRB levels suggests that E2F1 activity is tightly controlled in these cells. E2F1 acetylation enhances its transcriptional activity [21] but, unexpectedly, we found that E2F1 acetylation was induced by TNF-α, but not parthenolide. This indicates that enhanced acetylation might be a response of the cell to counteract the inhibition of E2F1 activity. Alternatively, it might be a consequence of the ability of IKK to potentiate E2F1 acetylation [22]. Consequently, parthenolide would then completely abrogate such acetylation.

NF-κB, and p65 in particular, is localized inside the nucleus also under basal conditions, where they may constitutively silence gene transcription by competing with other transcription factors [15]. The mechanism involved in the E2F1-mediated TNF-α downregulation of *PDK4* expression in AC16 cells entails enhanced physical interaction between the p65 subunit of NF-κB and E2F1. Importantly, this association was also observed in vivo in heart of TNF1.6 mice. Chromatin immunoprecipitation analyses demonstrated that NF-κB translocation to the nucleus prevented the recruitment of E2F1 to the *PDK4* promoter and its subsequent E2F1-mediated gene transcription. This view is supported by two observations: E2F1 binding to the *PDK4* promoter was reduced in TNF-α-treated cells; and parthenolide prevented this reduction. Importantly, parthenolide not only prevented the binding between p65-E2F1 by reducing translocation of the former into the nucleus, but would also activate E2F1 through enhanced pRB phosphorylation, thus releasing the active E2F-DP complex. This latter mechanism might account for the upregulation of *PDK4* expression induced by parthenolide in the absence of a proinflammatory stimulus. The association between p65 and E2F1 has already been established in human fibroblasts, in which this physical interaction disrupts the E2F-responsive gene expression [16]. Likewise, E2F1 has been reported to disrupt antiapoptotic NF-κB signaling through downregulation of the NF-κB activator TNF receptor-associated factor 2 [23], [24], by competing with p50 for RelA/p65 binding in murine fibroblasts [24] or inhibiting the phosphorylation of IκB [25]. In human AC16 cardiac cells, IL-6 expression and secretion was further induced after *E2F1* gene silencing, but downregulated by E2F1 overexpression. This indicates that E2F1 in these cells acts as a repressor of NF-κB activity. Although we only demonstrate the physical association of E2F1 with p65, it is feasible that other NF-κB subunits interact with this transcription factor, since other studies have revealed that exogenous E2F1 can associate with both RelA and p50 upon LPS stimulation [26]. The occurrence of additional mechanisms involving other E2Fs on the effects induced by NF-κB activation cannot be excluded either. Thus, it has been reported that IKKs may directly phosphorylate E2F4 in human fibroblasts. This results in nuclear accumulation of E2F4 and subsequent replacement of the activator E2F1 at the E2F-binding element in responsive gene promoters [16].

In the myocardium, glucose is catabolized predominantly by the aerobic glycolytic pathway [27]. PDK4 is the kinase responsible for the phosphorylation-induced inactivation of the pyruvate dehydrogenase complex, which catalyzes the rate-limiting step of aerobic glucose oxidation. Under certain circumstances, such as cardiac hypertrophy or heart failure, reliance on the glycolytic pathways is increased due to the downregulation of PDK4 activity. Based on our findings, we envision a model for the regulation of *PDK4* expression and cardiac cell metabolism by NF-κB and E2F1, in which NF-κB serves as a molecular switch that regulates E2F1-dependent *PDK4* gene transcription. As inappropriate PDK4 activity would have catastrophic consequences in high-metabolic-rate organs, the basal repression of E2F1-dependent *PDK4* expression by NF-κB might be crucial for normal cardiac function. Since E2F1 plays an important role in cardiac myocyte growth and is also involved in metabolism regulation through *PDK4* modulation, targeting this transcription factor could provide us with an effective therapy for treating detrimental left ventricular hypertrophy leading to heart failure. This is of particular relevance, since cardiac hypertrophy and chronic heart failure have both been related to inflammatory processes in the myocardium and we have also demonstrated that triggering E2F1 in human cardiac cells partially abrogates cytokine secretion.

## Methods

### Reagents

D-[U-^14^C]-glucose, [α-^32^P]-dATP and [γ-^32^P]-ATP were purchased from PerkinElmer (Waltham, MA, USA). All chemicals, except when indicated, were purchased from Sigma-Aldrich Química (Madrid, Spain). The antibodies utilized throughout the study were purchased from Cell Signaling Technology (Danvers, MA, USA), except Oct-1 and p65, which were from Santa Cruz Biotechnology (Inc., Heidelberg, Germany), and Lamin B (from Sigma-Aldrich Química).

### Cell culture and transient transfection studies

Human cardiac AC16 cells were maintained and grown as previously described [28]. In brief, non-differentiated AC16 cells were maintained in medium composed of Dulbecco's modified Eagle's medium (DMEM):F12 (Invitrogen, Barcelona, Spain) supplemented with 12.5% foetal bovine serum (FBS), 1% penicillin-streptomycin and 1% Fungizone (Invitrogen), and grown at 37°C in a humid atmosphere of 5% CO_2_/95% air until they reached 70–80% confluence. For in vitro overexpression studies, cells were transfected with Lipofectamine 2000 in OPTI-MEM reduced serum medium, following the manufacturer's recommendations (Invitrogen). For in vitro overexpression studies, the constructs used were pSG5L/E2F1 construct (human gene, Addgene plasmid 10736, Cambridge, MA, USA) [6] and the corresponding *LacZ*-carrying plasmid as a control. Transfection time and the DNA to Lipofectamine ratio for overexpression studies were set after optimization with the corresponding *LacZ*-carrying plasmid and using a β-galactosidase reporter gene staining kit (Sigma-Aldrich Química). Small interfering RNA (siRNA)-mediated *E2F1* gene silencing was carried out by transfecting AC16 cells with human *E2F1* siRNA (Santa Cruz Biotechnology), using scrambled siRNA as a transfection control. Fluorescein-labeled siRNA was used to optimize siRNA transfections by means of fluorescence microscopy.

### Preparation of cardiac samples from TNF-α transgenic mice

We used transgenic male TNF1.6 mice (8 to 12 –weeks old) with cardiac-specific overexpression of TNF-α, which has been established as a suitable model of cytokine-induced cardiomyopathy [29]. Mice were anaesthetized with 5% isoflurane and, after monitoring the adequacy of anaesthesia by testing of rear foot reflexes, they were euthanized by cervical dislocation. After this, the heart was excised, rinsed in ice-cold PBS and snap-frozen in liquid nitrogen [30]. The study was approved by the Thomas Jefferson University's Institutional Animal Care and Use Committee and conformed to the *Guide for the Care and Use of Laboratory Animals* published by the US National Institutes of Health (NIH Publication No. 85-23, revised 1996).

### RNA preparation and analysis

Relative levels of specific mRNAs were assessed by the reverse transcription-polymerase chain reaction (RT-PCR) [31]. Briefly, total RNA was isolated using the Ultraspec reagent (Biotecx, Houston, TX). RNA samples were cleaned (NucleoSpin RNA II; Macherey-Nagel, Düren, Germany) and checked for integrity by agarose gel electrophoresis. The total RNA isolated by this method was undegraded and free of protein and DNA contamination. Reverse transcription was performed from 0.5 µg total RNA using Oligo(dT)_23_ and M-MLV Reverse Transcriptase (Invitrogen). Preliminary experiments were carried out with various amounts of cDNA to determine nonsaturating conditions of PCR amplification for all the genes studied. Therefore, under these conditions, relative quantification of mRNA was assessed by the RT-PCR method described in this study [32]. Radioactive bands were quantified by video-densitometric scanning. The results for the expression of specific mRNAs are always presented relative to the expression of the control gene. The sequences of the forward and reverse primers used for amplification are shown in Supplementary Table S1.

### Electrophoretic mobility shift assay (EMSA) and immunoblot analysis

Nuclear extracts (NE) from AC16 cells were isolated as previously reported [31]. To obtain total proteins, AC16 cardiac cells or frozen tissue slides were homogenized in cold lysis buffer (5 mM Tris-HCl, pH 7.4, 1 mM EDTA, 0.1 mM phenylmethylsulfonyl fluoride, 1 mM sodium orthovanadate, and 5.4 µg/mL aprotinin). The homogenate was centrifuged at 10,000g for 30 min at 4°C. Protein concentration was determined using the Bradford method [33].

Electrophoretic mobility shift assay (EMSA) were performed using double-stranded oligonucleotides for the consensus binding site of E2F1 (Santa Cruz Biotechnology). Oligonucleotides were labeled by incubating the following reaction at 37°C for 2 hours: 2 µL oligonucleotide (1.75 pmol/µL), 2 µL of 5× kinase buffer, 1 µL of T4 polynucleotide kinase (10 U/µL), and 2.5 µL [γ-^32^P] ATP (3,000 Ci/mmol at 10 mCi/mL). The reaction was stopped by adding 90 µL of TE buffer (10 mM Tris-HCl, pH 7.4, and 1 mM EDTA). To separate the labeled probe from the unbound ATP, the reaction mixture was eluted in a Nick column (Pharmacia, Sant Cugat, Spain) according to the manufacturer's instructions. Five micrograms of crude nuclear protein were incubated for 10 min on ice in binding buffer (10 mM Tris-HCl, pH 8.0, 25 mM KCl, 0.5 mM dithiothreitol, 0.1 mM EDTA, pH 8.0, 5% glycerol, 5 mg/mL BSA, and 50 µg/ml poly[dI-dC]) in a final volume of 15 µL. Then, specific competitor oligonucleotide or antibody for supershift assays were added and incubated for 15 minutes on ice. Subsequently, the labeled probe (100,000 cpm) was added and the reaction was incubated for an additional 15 minutes on ice. Finally, protein-DNA complexes were resolved by electrophoresis at 4°C on 5% polyacrylamide gels in 0.5× Tris-borate-EDTA buffer and subjected to autoradiography.

Protein extracts were separated by SDS-PAGE on 10% separation gels and transferred to Immobilon polyvinylidene diflouride membranes (Millipore, Bedford, MA), as previously described [31]. Detection was achieved using the EZ-ECL chemiluminescence detection kit (Biological Industries, Beit Haemek, Israel). The size of detected proteins was estimated using protein molecular mass standards (Invitrogen).

### Coimmunoprecipitation and Chromatin immunoprecipitation (ChIP) studies

For coimmunoprecipitation, cell nuclear extracts (25 µg) were brought to a final volume of 250 µl with buffer containing 10 mM PBS, 50 mM KCl, 0.05 mM EDTA, 2.5 mM MgCl_2_, 8.5% glycerol, 1 mM dithiothreitol, 0.1% Triton X-100, BSA 2% and 1 mg/ml nonfat milk for 6 hours at 4°C and incubated with 4 µg of anti-p65. Immunocomplex was captured by incubating the samples with 50 µl protein A–agarose suspension (Santa Cruz Biotechnology) overnight at 4°C on a rocker platform. Agarose beads were collected by centrifugation and washed three times with PBS containing protease inhibitors. After microcentrifugation, the pellet was washed with 25 µl of SDS-PAGE sample buffer and boiled for 5 min at 100°C. The resultant supernatant was subjected to electrophoresis on 10% SDS-PAGE and immunoblotted with the corresponding antibodies.

Chromatin immunoprecipitation (ChIP) was performed with the ChIP Kit (Abcam, Cambridge, UK), using an E2F1-specific antibody. Input DNA, mock samples and E2F1-immunoprecipitated DNA were subjected to real-time PCR analysis with sequence-specific primers surrounding the E2F1 sites in the PDK4 promoter, along with primers capable of amplifying the genomic sequence lacking E2F binding sites (negative control, data not shown) [11]. Mock immunoprecipitations correspond to control reactions lacking antibodies. Results are reported as E2F1 site fold enrichment, expressed as a percentage of control samples ±STD, after normalization to the sample specific background and to the total input DNA.

### Glucose oxidation

AC16 cells were grown in 6-well plates as described above. Following transfection, 1 mL of reaction mixture (25 mmol/L NaHCO_3_, pH 7.4, 1.2 mmol/L MgSO_4_, 0.5 mmol/L CaCl_2_, 10 mmol/L HEPES, 1 µCi/mL D-[U-^14^C]-glucose) containing 1 µmol/L insulin was added to each well. After incubation for 60 minutes at 37°C in a water bath with gentle shaking, reactions were stopped by the injection of 100 µL 60% (w∶v) perchloric acid, and the plates were kept at 4°C overnight to trap the CO_2_ produced. The release of ^14^CO_2_ from glucose was measured by scintillation counting of the filter paper for 1 minute in a β-counter. Wells containing no cells were used as blanks. Glucose oxidation rates were calculated as nanomoles of added glucose·g^−1^ total protein total·hour^−1^, and then expressed as a percentage with respect to control cells.

### Statistical Analysis

Results are expressed as the mean ±SD of at least three separate experiments. Significant differences were established by either the Student's t test or one-way ANOVA, according to the number of groups compared, using the computer program GraphPad Prism (GraphPad Software Inc V4.03, San Diego, CA, USA). When significant variations were found by one-way ANOVA, the Tukey-Kramer multiple comparisons post-test was performed.

## Supporting Information

Figure S1 **E2F1 levels are not modified after NF-κB modulation.** (**A**) Relative quantification of *E2F1* mRNA levels assessed by RT-PCR in human cardiac AC16 cells incubated with TNF-α (100 ng/mL) for 6 h in the presence or absence of parthenolide (Parth, 10 µmol/L). The graphics represent the quantification of the *18S*-normalized mRNA levels, expressed as a percentage of control samples ±STD. (**B**) E2F1 protein levels in nuclear protein extracts isolated from samples as described in panel A. To show equal loading of protein, the Lamin B signal is also included. All autoradiograph data are representative of three separate experiments.
(TIF)

Figure S2 **NF-κB activation in transgenic TNF1.6 mice does not modulate E2F1 levels.** (**A**) Relative quantification of *PDK4*, *E2F1* and *Cyclin A* mRNA levels assessed by RT-PCR in left ventricle tissue of transgenic TNF1.6 and control wild-type (WT) mice. Graphs represent the quantification of the *Aprt*-normalized mRNA levels, expressed as a percentage of control samples ±STD. (**B**) E2F1 protein levels in nuclear protein extracts isolated from samples as described in panel **A**. To show equal loading of protein, the Lamin B signal is also included. The graphics represent the quantification of the normalized protein levels, expressed as a percentage of control samples ±STD. All autoradiograph data are representative of three separate experiments. *P<0.05, **P<0.01, and ***P<0.001 vs. WT
(TIF)

Table S1 Primers used for the RT-PCR reactions.
(DOC)
