# Expression of Concern: The Interplay between NF-kappaB and E2F1 Coordinately Regulates Inflammation and Metabolism in Human Cardiac Cells

**DOI:** 10.1371/journal.pone.0216434

**Published:** 2019-04-30

**Authors:** The PLOS ONE Editors

After publication of this article [1], concerns were raised about similarities between the following figure panels:

- 18S RT-PCR data in Fig 2A, Fig 3, Fig 6B, and horizontally flipped Fig 6A
- PDK4 data in Fig 1A lanes 1, 3, 4, 5, 6, and Fig 2A lanes 1, 5, 6, 4, 3, respectively

The authors noted that the 18S RT-PCR data are appropriately the same in Figs 3 and 6B, as they correspond to the same samples and experiments. Likewise, the authors clarified that the 18S RT-PCR panels in Figs 2A and 6A correspond to the same experiments, and that the correct 18S data for Figs 2A and 6A were presented in Fig 1A. The correct 18S data for Fig 1A are not available. The authors proposed that Cyclin A mRNA expression could be used as an alternate control for the PDK4, E2F1, IL-6 experiments as this did not appear to be affected by experimental treatments.

Regarding PDK4 data, the authors clarified that the wrong image is shown in Fig 1A, and that the results are correctly reported in Fig 2A. The authors explained that the Fig 1A PDK4 experiment replicates results reported previously in [2]. The PDK4 image reported in Fig 2A was spliced after lanes 4 and 6 to remove irrelevant data and rearrange lanes. The original full-size image for this panel is provided below in S1 File. The E2F1 panel in Fig 2A was spliced after lane 4, and the data shown in lane 7 of this figure appear to correspond to results for E2F1 alone rather than E2F1+TNF-α transfections, according to the original gel image shown in S1 File.

The original image data supporting Figs 1A, 2A, 3, 6A, and 6B are no longer available, except for the images underlying PDK4 and E2F1 results in Fig 2A (S1 File). The authors confirmed that data were quantified using the correct 18S controls corresponding to each experimental result, and that the image errors noted above do not impact the quantitative results reported in the article. Partial data tables were provided for the quantitative results in S1 File; these data tables do not include all experimental replicates for which results are summarized in the published graphs.

In light of the concerns raised concerning multiple figure panels, and in the absence of image data supporting some of the results, the *PLOS ONE* Editors issue this Expression of Concern.

The authors apologize for the errors in the published article.

## Supporting information

S1 File Underlying data file. Original image data supporting PDK4 and E2F1 results in Fig 2A, and quantitative data supporting graphs in Figs 1A, 2A, 3, 6.
(PDF)
