# Supplementary figures and images for: The Interplay between NF-kappaB and E2F1 Coordinately Regulates Inflammation and Metabolism in Human Cardiac Cells
Source: PLoS One. 2011 May 23;6(5):e19724. doi: 10.1371/journal.pone.0019724 (PMC3100304; doi:10.1371/journal.pone.0019724)

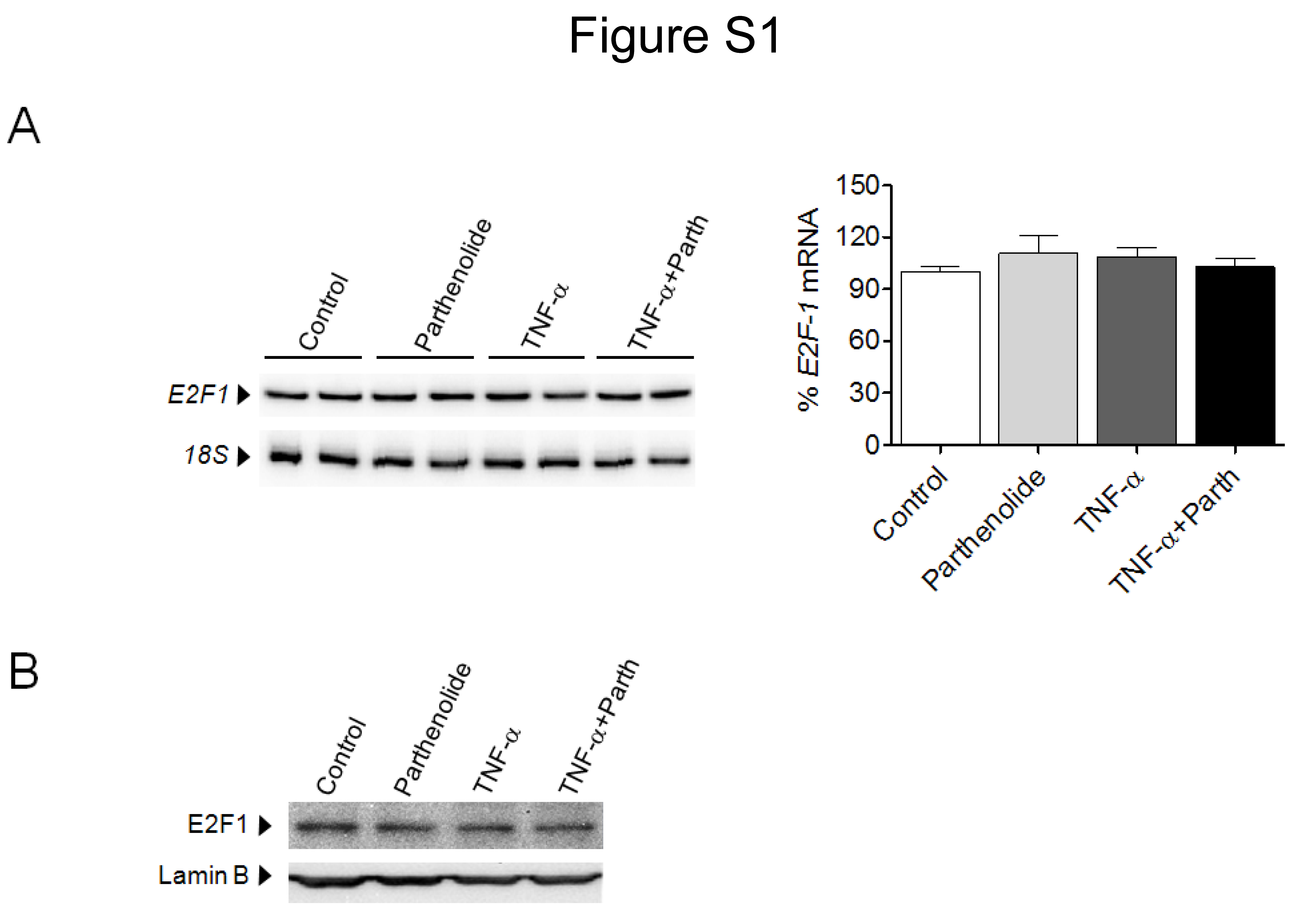

Supplement: Figure S1 — E2F1 levels are not modified after NF-κB modulation. (A) Relative quantification of E2F1 mRNA levels assessed by RT-PCR in human cardiac AC16 cells incubated with TNF-α (100 ng/mL) for 6 h in the presence or absence of parthenolide (Parth, 10 µmol/L). The graphics represent the quantification of the 18S-normalized mRNA levels, expressed as a percentage of control samples ±STD. (B) E2F1 protein levels in nuclear protein extracts isolated from samples as described in panel A. To show equal loading of protein, the Lamin B signal is also included. All autoradiograph data are representative of three separate experiments. (TIF) [file pone.0019724.s001.tif]

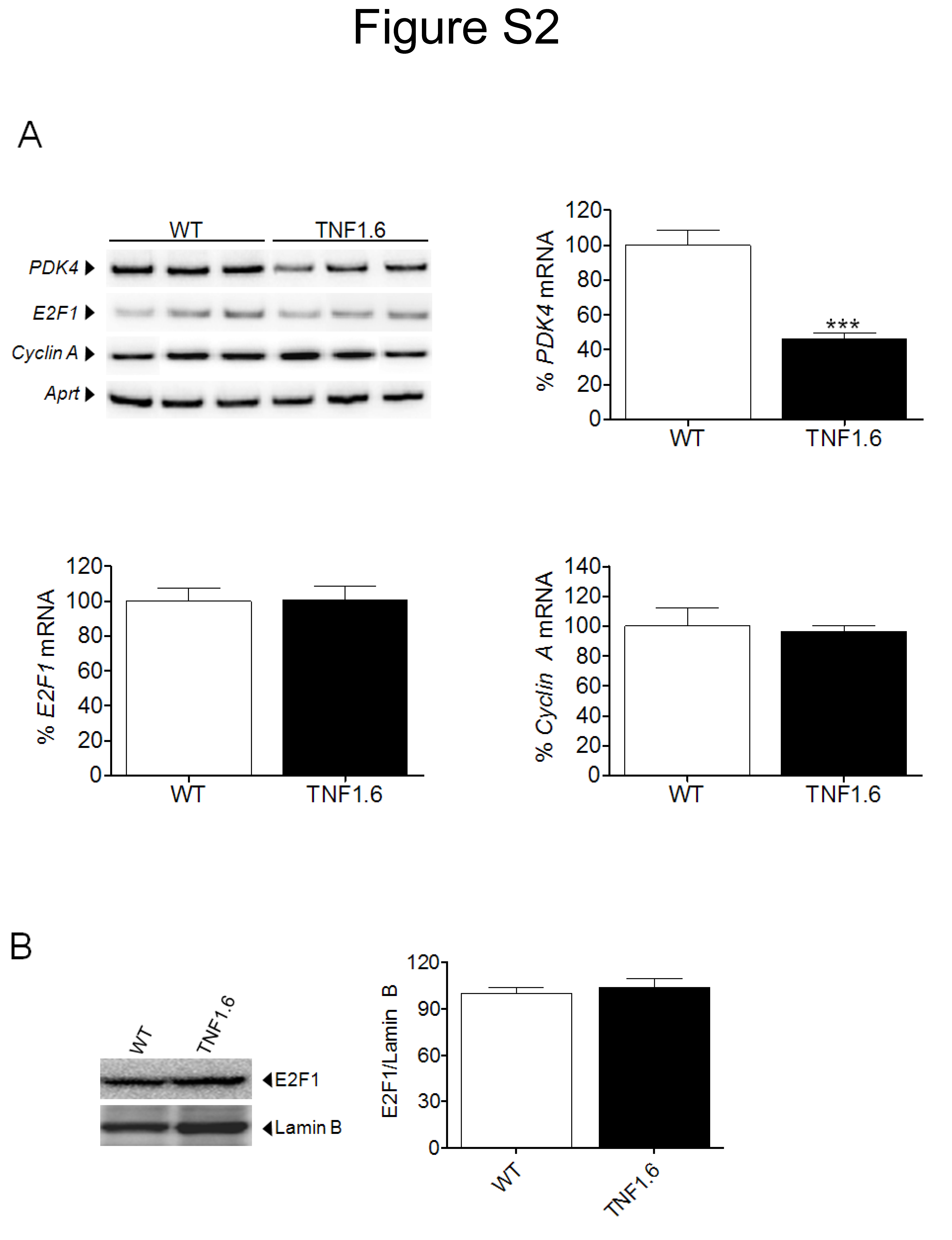

Supplement: Figure S2 — NF-κB activation in transgenic TNF1.6 mice does not modulate E2F1 levels. (A) Relative quantification of PDK4, E2F1 and Cyclin A mRNA levels assessed by RT-PCR in left ventricle tissue of transgenic TNF1.6 and control wild-type (WT) mice. Graphs represent the quantification of the Aprt-normalized mRNA levels, expressed as a percentage of control samples ±STD. (B) E2F1 protein levels in nuclear protein extracts isolated from samples as described in panel A. To show equal loading of protein, the Lamin B signal is also included. The graphics represent the quantification of the normalized protein levels, expressed as a percentage of control samples ±STD. All autoradiograph data are representative of three separate experiments. *P<0.05, **P<0.01, and ***P<0.001 vs. WT (TIF) [file pone.0019724.s002.tif]
