# Supplementary material for: The Interplay between NF-kappaB and E2F1 Coordinately Regulates Inflammation and Metabolism in Human Cardiac Cells
Source: PLoS One. 2011 May 23;6(5):e19724. doi: 10.1371/journal.pone.0019724 (PMC3100304; doi:10.1371/journal.pone.0019724)
Supplement: Table S1 — Primers used for the RT-PCR reactions. (DOC) [file pone.0019724.s003.doc]

**Supplemental Table**

**Table S1.** Primers used for the RT-PCR reactions.

| Gene | Forward primers | Reverse primers |
| --- | --- | --- |
| **Mice** |  |  |
| *APRT* | 5’-CAGCGGCAAGATCGACTACA-3’ | 5’-AGCTAGGGAAGGGCCAAACA-3’ |
| *Cyclin A* | 5’-CCCGCAGCAAGAAAACCACTG -3’ | 5’- TCCCACCTCAACCAGCCAGTC-3’ |
| *E2F1* | 5’- ACCTTCGCAGCATTGCAGACC-3’ | 5’-GCTGGGCCAGAGTCTGCAGTC -3’ |
| *PDK4* | 5’-AGGTCGAGCTGTTCTCCCGCT-3’ | 5’-GCGGTCAGGCAGGATGTCAAT-3’ |
| **Human** |  |  |
| *18S* | 5’-GCCGCTAGAGGTGAAATTCTTG-3’ | 5’-CATTCTTGGCAAATGCTTTCG-3’ |
| *Cyclin A* | 5’-TGCTAGCATTGCAGCAGACGG -3’ | 5’- CCTTAAGGGGTGCAACCCGTC-3’ |
| *E2F1* | 5’- TCAAAGCCCCTCCTGAGACCC-3’ | 5’- GGGGAGATGATGGTGGTGGTG-3’ |
| *IL-6* | 5’-CCCCCAGGAGAAGATTCCAA-3’ | 5’-TCAATTCGTTCTGAAGAGGTGAGT-3’ |
| *MCP-1* | 5’-GCTGTGATCTTCAAGACCATTGTG-3’ | 5’-TGGAATCCTGAACCCACTTCTG-3’ |
| *PDK4* | 5’-ACCCAAGCCACATTGGAAGCA-3’ | 5’-AACTGTTGCCCGCATTGCATT-3’ |
