# Supplementary material for: Expression of Concern: The Interplay between NF-kappaB and E2F1 Coordinately Regulates Inflammation and Metabolism in Human Cardiac Cells
Source: PLoS One. 2019 Apr 30;14(4):e0216434. doi: 10.1371/journal.pone.0216434 (PMC6490955; doi:10.1371/journal.pone.0216434)
Supplement: S1 File — Original image data supporting PDK4 and E2F1 results in Fig 2A, and quantitative data supporting graphs in Figs 1A, 2A, 3, 6. (PDF) [file pone.0216434.s001.pdf]

FIGURE 1A

18S RT-PCR

Lane	Area (global)
1. Control	7782637,67
2. Control	8420099,88
3. Parthenolide (24h)	6932689,84
4. Parthenolide (24h)	6791811,55
5. TNFa (24h)	8112455,27
6. TNFa (24h)	9495379,44
7. TNFa (24h) + Parthenolide	8386133,08
8. TNFa (24h) + Parthenolide	7092859,94

PDK4 RT-PCR

Lane	Area (global)	PDK4/18S	% vs. control
1. Control	5699145,72	0,7323	**102,61**
2. Control	5852153,65	0,6950	**97,39**
3. Parthenolide (24h)	8570159,09	1,2362	**173,22**
4. Parthenolide (24h)	6852098,60	1,0089	**141,37**
5. TNFa (24h)	987906,45	0,1218	**17,06**
6. TNFa (24h)	1149845,84	0,1211	**16,97**
7. TNFa (24h) + Parthenolide	4461940,42	0,5321	**74,55**
8. TNFa (24h) + Parthenolide	6333748,38	0,8930	**125,13**

E2F1 RT-PCR

Lane	Area (global)	E2F1/18S	% vs. control
1. Control	217770,56	0,0280	**113,20**
2. Control	180671,11	0,0215	**86,80**
3. Parthenolide (24h)	181429,97	0,0262	**105,87**
4. Parthenolide (24h)	151806,96	0,0224	**90,42**
5. TNFa (24h)	139264,16	0,0172	**69,45**
6. TNFa (24h)	207865,50	0,0219	**88,56**
7. TNFa (24h) + Parthenolide	196173,37	0,0234	**94,63**
8. TNFa (24h) + Parthenolide	179780,41	0,0253	**102,54**

FIGURE 2A

PDK4 RT-PCR

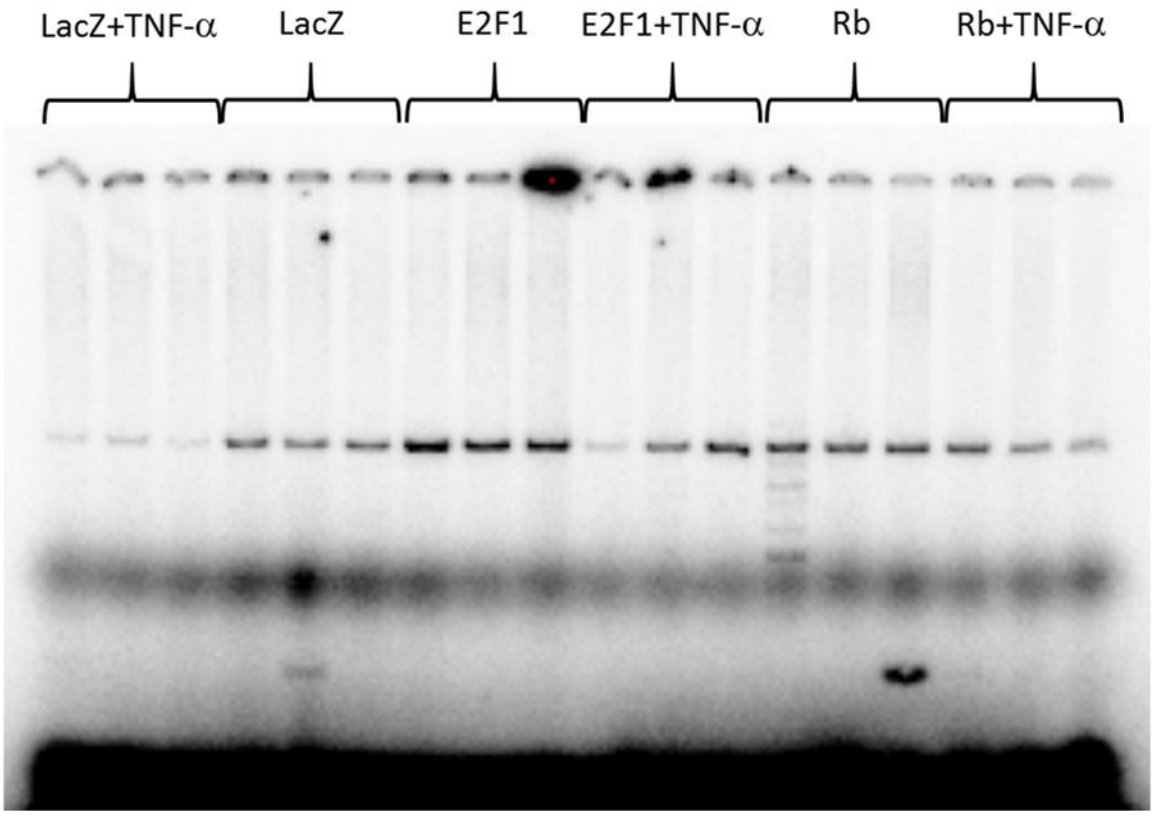

E2F1 RT-PCR

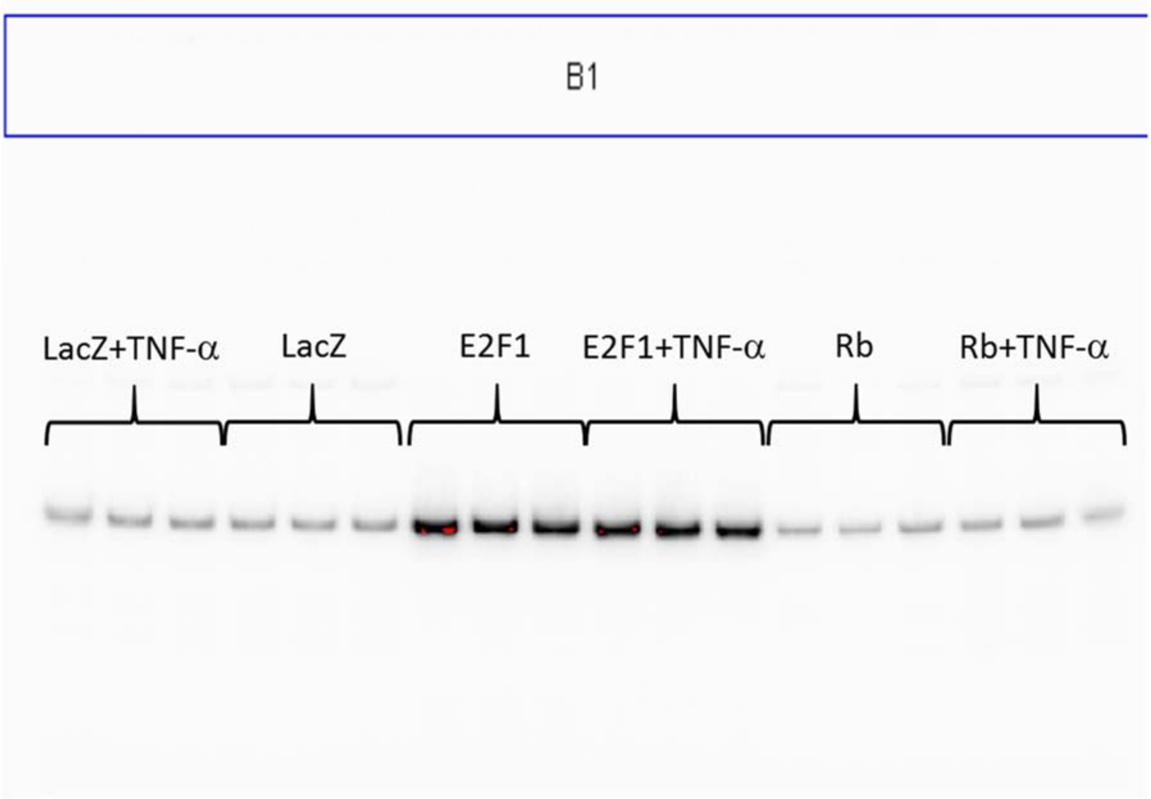

18S RT-PCR

Lane	Area (global)
1. LacZ	179736,5619
2. LacZ	196972,8024
3. LacZ+TNFα 100ng/ml	194715,2024
4. LacZ+TNFα 100ng/ml	183926,9621
5. E2F-1	176393,2818
6. E2F-1	178316,0419
7. E2F-1+TNFα 100ng/ml	150537,2411
8. E2F-1+TNFα 100ng/ml	153927,1612

E2F1 RT-PCR

Lane	Area (global)	E2F1/18S
1. LacZ	139720,68	0,7774
2. LacZ	140170,08	0,7116
3. LacZ+TNFα 100ng/ml	133041,52	0,7233
4. LacZ+TNFα 100ng/ml	159328,20	0,8663
5. E2F-1	633492,94	3,5914
6. E2F-1	520857,37	2,9210
7. E2F-1+TNFα 100ng/ml	476186,21	3,1632
8. E2F-1+TNFα 100ng/ml	526673,13	3,4216

PDK4 RT-PCR

Lane	Area (global)	PDK4/18S
LacZ A2	34660,45	0,1760
LacZ A3	37759,21	0,1939
LacZ+TNFα 100ng/ml B1	12030,81	0,0618
LacZ+TNFα 100ng/ml B2	15902,69	0,0865
E2F-1 C1	83647,34	0,4742
E2F-1 C2	67307,53	0,3775
E2F-1+TNFα 100ng/ml D1	36545,97	0,2428
E2F-1+TNFα 100ng/ml D2	53589,29	0,3481

FIGURE 3

18S RT-PCR

Lane	Area (global)
1. siRNA Ctrl	146210,6012
2. siRNA Ctrl	147582,7214
3. siRNA Ctrl+TNFα	161593,7628
4. siRNA Ctrl+TNFα	154657,8421
5. siE2F1	147312,4372
6. siE2F1	144805,1211
7. siE2F1+TNFα	133007,4399
8. siE2F1+TNFα	126168,6791

E2F1 RT-PCR

Lane	Area (global)	E2F1/18S	% vs. control
1. siRNA Ctrl	679243,79	4,6457	**96,10**
2. siRNA Ctrl	741270,16	5,0227	**103,90**
3. siRNA Ctrl+TNFα	766674,94	4,7445	**98,14**
4. siRNA Ctrl+TNFα	698258,50	4,5149	**93,39**
5. siE2F1	337313,64	2,2898	**47,37**
6. siE2F1	410545,32	2,8352	**58,65**
7. siE2F1+TNFα	444250,45	3,3400	**69,09**
8. siE2F1+TNFα	483488,90	3,8321	**79,27**

PDK4 RT-PCR

Lane	Area (global)	PDK4/18S	% vs. control
1. siRNA Ctrl	46103,19	0,3153	**97,04**
2. siRNA Ctrl	49378,53	0,3346	**102,96**
3. siRNA Ctrl+TNFα	31018,79	0,1920	**59,07**
4. siRNA Ctrl+TNFα	24173,30	0,1563	**48,10**
5. siE2F1	46151,66	0,3133	**96,41**
6. siE2F1	44509,23	0,3074	**94,59**
7. siE2F1+TNFα	30686,07	0,2307	**71,00**
8. siE2F1+TNFα	28229,48	0,2237	**68,85**

Cyclin A RT-PCR

Lane	Area (global)	Cyclin/18S	% vs. control
1. siRNA Ctrl	106578,41	0,7289	**94,67**
2. siRNA Ctrl	119693,11	0,8110	**105,33**
3. siRNA Ctrl+TNFα	87876,99	0,5438	**70,63**
4. siRNA Ctrl+TNFα	145892,29	0,9433	**122,51**
5. siE2F1	88705,55	0,6022	**78,20**
6. siE2F1	110301,39	0,7617	**98,93**
7. siE2F1+TNFα	89851,13	0,6755	**87,73**
8. siE2F1+TNFα	127168,63	1,0079	**130,90**

FIGURE 6A

18S RT-PCR 15-10-09,
Gel 38/09 (16-10-09)

Lane	Area (global)
1. LacZ	179736,5619
2. LacZ	196972,8024
3. LacZ+TNFα 100ng/ml	194715,2024
4. LacZ+TNFα 100ng/ml	183926,9621
5. E2F-1	176393,2818
6. E2F-1	178316,0419
7. E2F-1+TNFα 100ng/ml	150537,2411
8. E2F-1+TNFα 100ng/ml	153927,1612

IL-6 RT-PCR

Lane	Area (global)	IL-6/18S	% vs. control
LacZ A2	168059,20	0,9350	**103,97**
LacZ A3	170095,16	0,8635	**96,03**
LacZ+TNFα 100ng/ml B1	283530,05	1,4561	**161,92**
LacZ+TNFα 100ng/ml B2	303897,61	1,6523	**183,73**
E2F-1 C1	179379,88	1,0169	**113,08**
E2F-1 C2	184286,16	1,0335	**114,92**
E2F-1+TNFα 100ng/ml D1	248591,57	1,6514	**183,63**
E2F-1+TNFα 100ng/ml D2	201401,40	1,3084	**145,50**

FIGURE 6B

18S RT-PCR

Lane	Area (global)
1. siRNA Ctrl	146210,6012
2. siRNA Ctrl	147582,7214
3. siRNA Ctrl+TNFα	161593,7628
4. siRNA Ctrl+TNFα	154657,8421
5. siE2F1	147312,4372
6. siE2F1	144805,1211
7. siE2F1+TNFα	133007,4399
8. siE2F1+TNFα	126168,6791

IL-6 RT-PCR

Lane	Area (global)	IL-6/18S	% vs. control
1. siRNA Ctrl	279455,31	1,9113	**95,37**
2. siRNA Ctrl	309493,64	2,0971	**104,63**
3. siRNA Ctrl+TNFα	1228198,06	7,6005	**379,23**
4. siRNA Ctrl+TNFα	1149517,04	7,4326	**370,85**
5. siE2F1	362484,23	2,4606	**122,77**
6. siE2F1	354216,34	2,4462	**122,05**
7. siE2F1+TNFα	1490262,11	11,2044	**559,04**
8. siE2F1+TNFα	1263460,14	10,0141	**499,65**
